# Genetic Analysis of Peroxisomal Genes Required for Longevity in a Yeast Model of Citrin Deficiency

**DOI:** 10.3390/diseases8010002

**Published:** 2020-01-09

**Authors:** Chalongchai Chalermwat, Thitipa Thosapornvichai, Laran T. Jensen, Duangrurdee Wattanasirichaigoon

**Affiliations:** 1 Department of Biochemistry, Faculty of Medicine, Khon Kaen University, Khon Kaen 40002, Thailand; 2 Department of Biochemistry, Faculty of Science, Mahidol University, Bangkok 10400, Thailand; pa_lim_piim@hotmail.com (T.T.); laran.jen@mahidol.ac.th (L.T.J.); 3 Division of Medical Genetics, Department of Pediatrics, Faculty of Medicine Ramathibodi Hospital, Mahidol University, Bangkok 10400, Thailand; duangrurdee.wat@mahidol.ac.th

**Keywords:** citrin deficiency, mitochondrial aspartate–glutamate carrier, peroxisomes, NAD^+^ regeneration, *Saccharomyces cerevisiae*

## Abstract

Citrin is a liver-specific mitochondrial aspartate–glutamate carrier encoded by *SLC25A13*. Citrin deficiency caused by *SLC25A13* mutation results in carbohydrate toxicity, citrullinemia type II, and fatty liver diseases, the mechanisms of some of which remain unknown. Citrin shows a functional homolog in yeast aspartate-glutamate carrier (Agc1p) and *agc1*Δ yeasts are used as a model organism of citrin deficiency. Here, we found that *agc1*Δ yeasts decreased fat utilization, impaired NADH balance in peroxisomes, and decreased chronological lifespan. The activation of *GPD1*-mediated NAD^+^ regeneration in peroxisomes by *GPD1* over-expression or activation of the malate–oxaloacetate NADH peroxisomal shuttle, by increasing flux in this NADH shuttle and over-expression of *MDH3*, resulted in lifespan extension of *agc1*Δ yeasts. In addition, over-expression of *PEX34* restored longevity of *agc1*Δ yeasts as well as wild-type cells. The effect of *PEX34*-mediated longevity required the presence of the *GPD1*-mediated NADH peroxisomal shuttle, which was independent of the presence of the peroxisomal malate–oxaloacetate NADH shuttle and *PEX34*-induced peroxisome proliferation. These data confirm that impaired NAD^+^ regeneration in peroxisomes is a key defect in the yeast model of citrin deficiency, and enhancing peroxisome function or inducing NAD^+^ regeneration in peroxisomes is suggested for further study in patients’ hepatocytes.

## 1. Introduction

Citrin deficiency is an inherited non-alcoholic fatty liver disease commonly found in the Asia–Pacific population [1,2,3]. This disorder results from a loss-of-function mutation in *SLC25A13* encoding a mitochondrial aspartate–glutamate carrier, called citrin or AGC2, that appears to have a primary role in the liver [4,5]. Citrin deficiency results in an impaired malate–aspartate shuttle and decreased NAD^+^ regeneration in the cytosol leading to carbohydrate intolerance and type II citrullinemia (CTLN2) [6,7]. Current therapies for citrin deficiency are limited, although treatment with sodium pyruvate, arginine hydrochloride, and restriction of carbohydrate intake has shown promise in reducing symptoms [7,8].

*Saccharomyces cerevisiae* (budding yeast) has been shown to be a suitable model system for the study of several human diseases, including citrin deficiency [9,10]. *S. cerevisiae* is a genetically tractable organism and can be used to search for genetic suppressors to identify functional relationships between genes that may not otherwise be identified via other techniques [11]. Yeast contains a homolog of citrin, *AGC1*, and the deletion of this gene results in decreased growth in acetate and oleic media [12]. Ectopic expression of *SLC25A13* in *agc1*Δ yeasts can restore growth in acetate medium, demonstrating that yeast *AGC1* is functionally equivalent to human citrin [10,12].

In addition to mitochondria, peroxisomes can contribute to utilization of acetate and fatty acids [13,14,15]. Loss of *AGC1* in yeasts impairs growth in acetate and oleic media, but not in glycerol medium, suggesting that it likely affects peroxisomal rather than mitochondrial functions [10]. Peroxisomes are single-membrane-bound organelles derived from the endoplasmic reticulum (ER) and are found in eukaryotic cells including those of both yeasts and humans [16]. A major function of peroxisomes is to support mitochondrial metabolism through fatty acid oxidation as well as degradation of hydrogen peroxide, and these activities are induced in yeast following entry into the stationary phase of growth [16,17,18]. Survival of yeast in the stationary phase has been extensively utilized as a model of aging and is referred to as the chronological lifespan [19]. In this study, we report that *agc1*Δ yeast have impaired peroxisomal NAD^+^ regeneration, decreased fat utilization, and decreased chronological lifespan. Genetic analysis of *agc1*Δ yeast has revealed that the induction of peroxisome functions relating to NAD^+^ regeneration is capable of increasing the longevity of *agc1*Δ yeast. The reduced lifespan of human hepatocytes with a loss-of-function mutation in citrin may contribute to disease progression. Therefore, enhancing peroxisome function may be an alternative treatment for citrin deficiency.

## 2. Materials and Methods

### 2.1. Primers and Plasmids

The *AGC1* disruption plasmid was generated by PCR amplifying upstream (−931 to −114) and downstream sequences (+2499 to +3478) of *ACG1* introducing *Bam*HI and *Sal*I (upstream) or *Not*I and *Bam*HI (downstream) restriction sites. Following digestion, the *AGC1* DNA fragments were inserted into pRS403 (*HIS3*) [20]. The expression plasmids including *PEX34*, *PEX5*, *PEX11*, *MDH3*, *GPD1*, and *NDE2* expression plasmids were constructed using Ycplac33 (*CEN URA3*) vector, a yeast centromeric plasmid containing the *URA3* selectable marker and the *TPI1* promoter [21]. The NADH sensor plasmids were derived from pC1-REX-YFP, a gift from Vsevolod Belousov (Addgene plasmid #48247). The REX-YFP fusion protein will interact with NADH and can emit fluorescent signals [22]. pC1-REX-YFP was introduced at 5′ XbaI and NdeI sites by PCR mutagenesis and verified. The mutated pC1-REX-YFP was digested with XbaI and SacII to obtain the NADH sensor sequence. The backbone plasmid pLJ339 (*PGK1* promoter, *CEN*, *LEU2*), a yeast centromeric plasmid containing the *LEU2* selectable marker and the *PGK1* promoter, was digested with the same enzymes. The NADH sensor sequence was then ligated to the isolated pLJ339 backbone plasmid. The cytosolic NADH sensor plasmid was selected and verified. To construct the peroxisomal NADH sensor plasmid, the NADH sensor sequence in the cytosolic NADH sensor plasmid was introduced a C-terminal peroxisomal targeting sequence by PCR mutagenesis. The cytosolic NADH sensor plasmid was selected and verified. The lists of primers used in the study is shown in Supplementary Table S1.

### 2.2. Yeast Strains and Disruption of AGC1

Yeast strains used in this study were derived from BY4742 [23]. Single-deletion yeast strains were obtained from Open Biosystems, Inc. (Waltham, MA, USA). The *AGC1* gene was disrupted using the knockout plasmid, linearized by digestion with *Bam*H1, and transformed into yeast strains. The *AGC1* gene of yeast was replaced with the *HIS3* selectable marker by homologous recombination. Correct plasmid integration and deletion of *AGC1* sequences was verified by in vivo PCR using flanking primers [24]. The lists of yeast strains and genotypes used in the study is shown in Supplementary Table S2.

### 2.3. Yeast Transformation

The control plasmid (pRS316), expression plasmids, and linearized AGC1 disruption plasmids were transformed into specific yeast strains using a lithium acetate procedure [25]. Cells were propagated at 30 °C either in enriched yeast extract, peptone-based medium (YPD), synthetic complete (SC), or synthetic deficient (SD) medium containing 2% glucose [26]. Yeast transformants were selected on SC medium lacking the appropriate nutrient.

### 2.4. Culture Conditions and Longevity Assay

Yeasts were grown on synthetic deficient (SD) plate supplemented with 2% glucose incubated in a 30 °C incubator for three days. Then, they were pre-cultured in synthetic SD broth containing 2% glucose by incubation at 30 °C, with shaking at 220 rpm for 24 h. Cells were harvested and inoculated in SD broth with 2% glucose at a starting optical density 600 nm (OD_600_) of 0.1 and grown for nine days. The chronological longevity assay was used to determine survival in the stationary phase [19]. Cell survival was monitored at Day 3 and Day 9 of culture. Yeast cells were collected and washed twice with sterile distilled water. The OD_600_ was measured, and samples were prepared in sterile distilled water at 10^5^, 10^4^, and 10^3^ cells. Yeasts (10 µL each) were dropped on an SD plate supplemented with 2% glucose, incubated at 30 °C for 72 h, and then imaged.

### 2.5. Peroxisomal and Cytosolic NADH Analysis, Peroxisome Proliferation Analysis, and Confocal Fluorescence Microscopy

To analyze peroxisomal and cytosolic NADH fluorescence signals, yeasts were grown on an SD plate supplemented with 2% glucose incubated in a 30 °C incubator for five days and then directly taken for visualizing NADH signals with confocal fluorescence microscopy. The excitation and emission wavelengths were chosen at 488/510 nm. The small punctate green fluorescence represented peroxisomal NADH signals and the diffuse green fluorescence represented cytosolic NADH signals. To analyze peroxisome proliferation, yeasts were grown on an SD plate supplemented with 2% glucose for three days. Then, they were cultured in SD broth supplemented with 0.1% glucose until late log phase. The punctate signals from red fluorescent protein (RFP) fusions RFP-PTS1 (peroxisome marker) represented peroxisome numbers [27]. To visualize fluorescence signals derived from NADH sensor protein or RFP-PTS1 protein, the live cells were directly viewed at a magnification of 60× with an Olympus FV1000 confocal laser scanning microscope (Olympus Bioimaging Center, Mahidol University).

### 2.6. Neutral Lipid Staining and Spectrofluorometry

To analyze neutral lipids (esterified fatty acids), yeasts were precultured and cultured the same as for longevity assays. Cells were taken for staining neutral lipids with Nile red fluorescence dye at Day 3 and Day 9 of culture. One milliliter of cell volumes was collected, washed with phosphate buffered saline twice, and resuspended to OD_600nm_ 1.0 (2 × 10^4^ cell/µL). Then, 250 µL of cell volumes (5 × 10^6^ cells) was incubated with dimethyl sulfoxide: phosphate buffered saline 1:1 for 2 min in one well of a 96-well plate. Yeasts were then stained with 25 µL of Nile red (60 µg/mL). The fluorescence signals in 5 µg/mL of Nile red were read for 20 min (read every 1 min × 20 cycles) during shaking at 180 rpm at 30 °C in a spectrofluorometer machine. The excitation and emission wavelength were chosen at 485/535 nm. The experiment was performed in triplicate, and the highest fluorescence intensity prior to plateau was chosen for analysis. The relative fluorescence intensity was corrected with background fluorescence intensity obtained from the control well. Neutral lipid contents in yeasts were presented as relative fluorescence units.

### 2.7. Data Analysis

The difference in neutral lipid contents was analyzed by IBM SPSS statistics version 22. Statistical analyses within groups were done using paired-samples *t* tests (paired *t*-test) and a significant difference was indicated when *p*-value <0.05. Yeasts with neutral lipid staining were derived in triplicate from one repeated experiment.

## 3. Results

### 3.1. Deletion of AGC1 Inhibits Fat Utilization in the Stationary Phase

**Figure 1:**
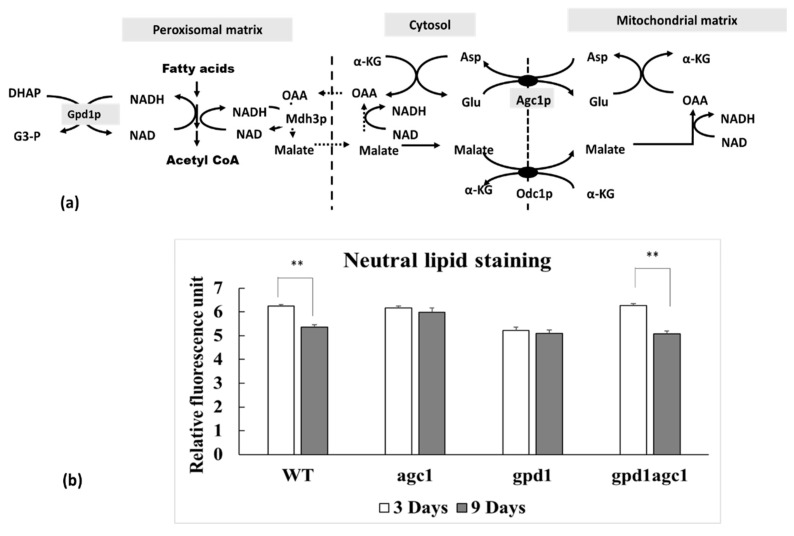
Role of mitochondrial aspartate–glutamate carrier (Agc1p) in the malate–aspartate shuttle involved in fatty acid oxidation in peroxisomes and alteration of fat utilization of *agc1*Δ yeasts and related yeast mutants in the stationary phase. (**a**) The malate–aspartate shuttle in yeasts is required for NAD^+^ regeneration in peroxisomes with the transfer of the reducing equivalent from peroxisome into mitochondria. Reducing equivalents generated in peroxisomes are derived from fatty acid oxidation which is enhanced by Mdh3p-mediated and Gpd1p-mediated NAD^+^ regeneration in peroxisomes. Note that both the malate–oxaloacetate NADH shuttle (dot line) and the malate–aspartate shuttle are linked for NAD^+^ regeneration in peroxisomes. (**b**) Wild-type and yeast mutants including *agc1*Δ, *gpd1*Δ, and *gpd1*Δ*agc1*Δ cells in stationary phase were selected for neutral lipid staining with Nile red fluorescence dye and then the fluorescence intensity was measured with spectrofluorometry. Note that deletion of *AGC1* decreased fat utilization, but deletion of *GPD1* decreased both of fat storage and utilization in the stationary phase. Deletion of *AGC1* in *gpd1*Δ yeasts restored fat storage and fat utilization in the stationary phase. Abbreviations: Asp, aspartate; Glu, glutamate; OAA, oxaloacetate; α-KG, α-ketoglutarate, Agc1p, aspartate–glutamate carrier, Odc1p, oxodicarboxylate carrier; Mdh3p, malate dehydrogenase3; Gpd1p, glycerol 3-phosphate dehydrogenase; DHAP, dihydroxyacetone phosphate; G3-P, glycerol 3-phosphate dehydrogenase. ** *p* ≤ 0.01.

Fatty acid is esterified and stored in fat droplets in cytosol as an energy reserve in yeast cells. It will be liberated from triacylglycerol and oxidized in peroxisomes when cells enter the stationary phase. Fatty acid oxidation requires plenty of NAD^+^ as a coenzyme which is continually supplied by Mdh3p and Gpd1p activity in peroxisomes (Figure 1a). Mdh3p is malate dehydrogenase localizing in peroxisomes and Gpd1p is glycerol 3-phosphate dehydrogenase localizing in both cytosolic and peroxisomal compartments [28]. The substrate for Mdh3p is oxaloacetate derived from aspartate transamination which is replenished by Agc1p activity (Figure 1a). As seen in Figure 1b, deletion of *AGC1* decreased fat utilization in the stationary phase, but deletion of *GPD1* decreased both of fat storage and fat utilization. Interestingly, deletion of *AGC1* in *gpd1*Δ yeasts restored the capability of fat storage and fat utilization in the stationary phase.

### 3.2. Deletion of AGC1 Increases Peroxisomal NADH Signals, but Decreases Cytosolic NADH Signals in the Stationary Phase

**Figure 2:**
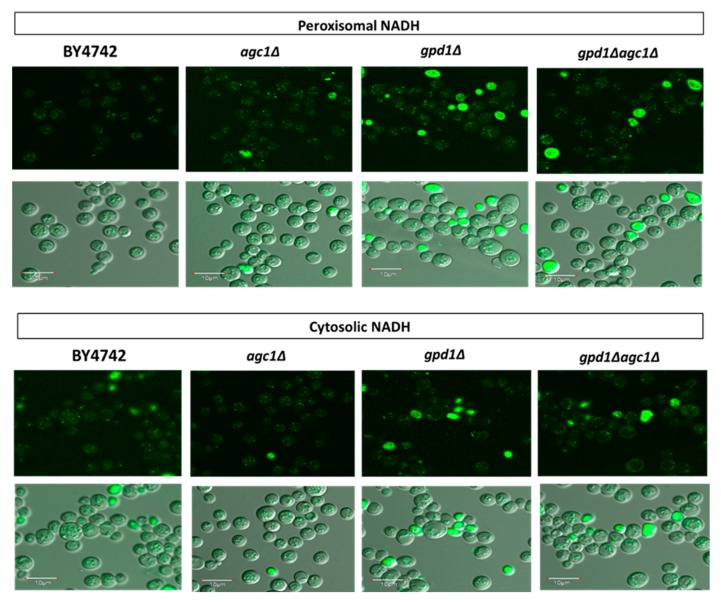
Deletion of *AGC1* increases peroxisomal NADH signals but decreases cytosolic NADH signals in the stationary phase. Yeasts containing peroxisomal and cytosolic NADH sensor plasmids were grown in a synthetic deficient (SD) medium plate supplemented with 2% glucose for five days and then taken for visualizing NADH signals with confocal fluorescence microscopy. The small punctate green fluorescence signals represented NADH localizing in peroxisomes. Note that deletion of *GPD1* showed similar effects as deletion of *AGC1*, and deletion of *AGC1* in *gpd1*Δ cells also restored cytosolic NADH signals. In addition, cytosolic NADH signals induced by peroxisomal NADH sensor plasmids and peroxisomal NADH signals induced by cytosolic NADH sensor plasmids were observed.

NAD^+^ regeneration in peroxisomes supplies NAD^+^ for fatty acid oxidation. To further explain whether impaired fat utilization in *agc1*Δ yeasts is due to decreased NAD^+^ regeneration in peroxisomes, we demonstrated peroxisomal NADH signals in yeasts by peroxisomal NADH sensor plasmids. As shown in Figure 2, increased peroxisomal NADH signals, which are represented as small punctate green fluorescence signals, were observed in *agc1*Δ, *gpd1*Δ, and *gpd1*Δ*agc1*Δ yeasts. Deletion of *GPD1* showed slightly higher peroxisomal NADH signal intensity relative to others. However, some NADH signals induced by peroxisome NADH sensor plasmids were also found in the cytoplasm. In addition, we used cytosolic NADH sensor plasmids to directly demonstrate cytosolic NADH signals which might be affected in *agc1*Δ and *gpd1*Δ yeasts. The results showed that the cytosolic NADH signals were decreased in *agc1*Δ and *gpd1*Δ yeasts, but these signals were restored in *gpd1*Δ*agc1*Δ yeasts. As seen in Figure 2, some NADH signals induced by cytosolic NADH sensor plasmids were also observed in peroxisomes.

### 3.3. Over-Expression of PEX34, MDH3, GPD1 Increases the Longevity of agc1Δ Yeasts in the Stationary Phase

Peroxisome functions are important for the stationary phase of cell growth, and deletion of *ACG1* impairs utilization of acetate and oleic acid, which are important carbon sources in the stationary phase [12,17,19]. Peroxisome biogenesis requires both proliferation of the peroxisome structure and import of peroxisomal proteins. *PEX34* and *PEX11* function in peroxisome proliferation [16]. In contrast, *PEX5* functions in the import of peroxisome matrix proteins, such as Mdh3p, which is important for NAD^+^ regeneration in peroxisomes [16,28].

**Figure 3:**
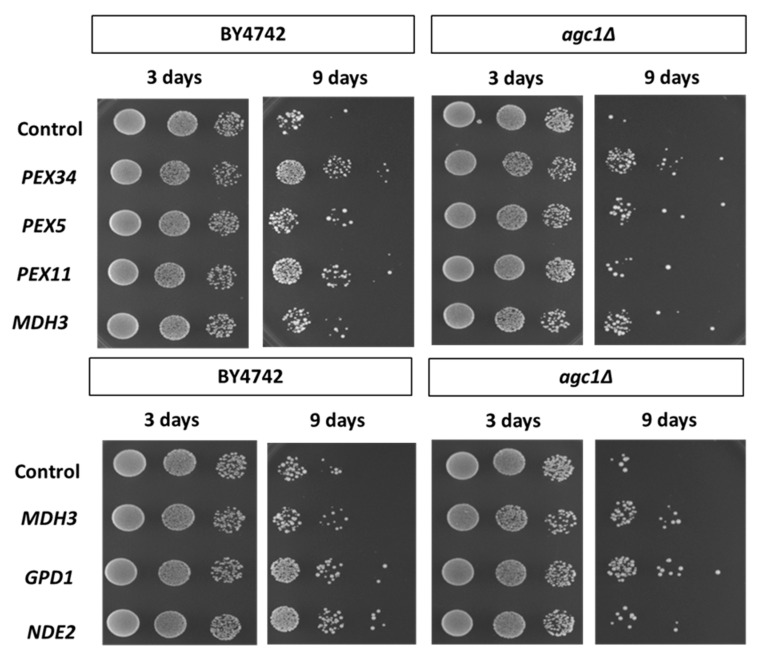
Effects of over-expression of peroxisomal genes and NAD^+^ regeneration genes on the chronological longevity of *agc1*Δ yeasts. Wild-type and *agc1*Δ yeasts containing expression plasmids of peroxisomal genes and NAD^+^ regeneration genes as indicated were cultured in SD broth supplemented with 2% glucose and tested for chronological longevity. The yeast array on an SD plate supplemented with 2% glucose indicated viability starting with 10^5^, 10^4^, and 10^3^ cells. Note that *agc1*Δ yeasts had decreased chronological longevity, and *GPD1*, *MDH3*, and *PEX34* over-expression increased the longevity of *agc1*Δ yeasts.

As seen in Figure 3, deletion of *AGC1* decreased the chronological lifespan of yeast cells. Over-expression of Pex34p, Pex5p, and Mdh3p in *agc1*Δ cells increased lifespan, but the over-expression of Pex34p, and Pex11p increased longevity in wild-type cells. Interestingly, over-expression of Gpd1 in *agc1*Δ cells also increased the chronological lifespan and over-expression of Nde2p, which is an external NADH dehydrogenase in the mitochondria required for catalyzing NADH in cytosol [29], failed to restore the chronological lifespan of *agc1*Δ cells. Conversely, over-expression of Nde2p and Gpd1p increased the chronological lifespan of wild-type yeasts.

### 3.4. PEX34-Mediated Chronological Longevity Requires GPD1 but not PEX25 and PEX27

**Figure 4:**
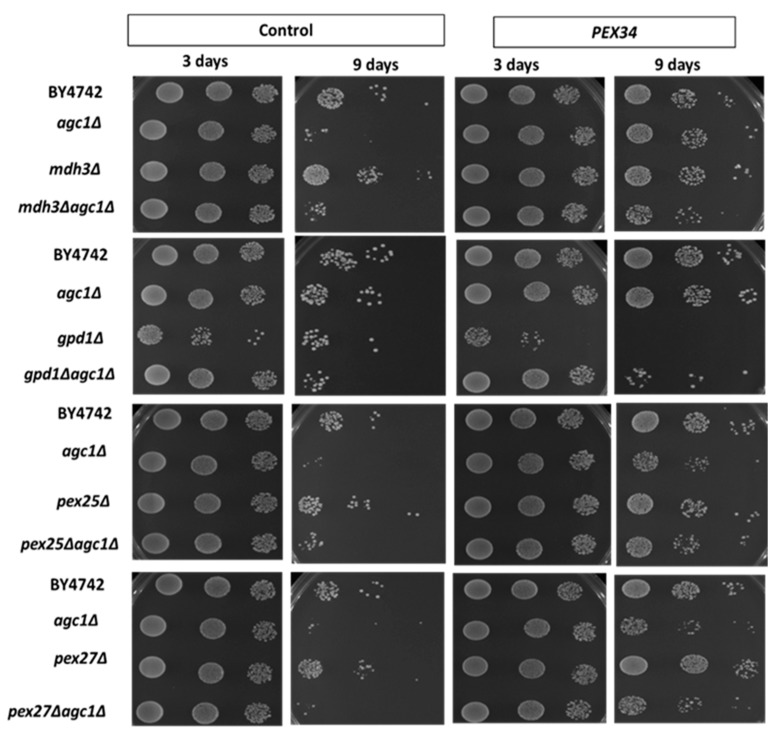
*PEX34*-mediated longevity requires peroxisomal genes for NAD^+^ regeneration, but not for peroxisome proliferation. Yeasts lacking genes involved in NAD^+^ regeneration in the peroxisome (*MDH3*, *GPD1*) and peroxisome proliferation (*PEX25*, *PEX27*) were tested for chronological longevity under *PEX34* over-expression. The yeast array on an SD plate supplemented with 2% glucose indicated viability starting with 10^5^, 10^4^, and 10^3^ cells. Note that *PEX34*-mediated longevity requires *GPD1* expression and loss of *GPD1* decreased the longevity at the early stationary phase.

We have known that the induction of NAD^+^ regeneration in peroxisomes was a primary mechanism of chronological longevity in *agc1*Δ yeasts. To determine whether *MDH3* was important for increased longevity from over-expression of *PEX34*, we examined the lifespan of an *agc1*Δ*mdh3*Δ strain. The increased chronological lifespan in *agc1*Δ cells from *PEX34* over-expression was still apparent, although reduced, when *MDH3* was also deleted (Figure 4). This indicates that while over-expression of *MDH3* can extend yeast lifespan, this gene does not have a major role in lifespan extension from *PEX34* over-expression. In addition, we determined whether *GPD1* was important for promoting longevity in *agc1*Δ yeasts. Interestingly, *PEX34* over-expression in the *gpd1*Δ strain caused decreased longevity; however, the negative effect of *PEX34* was abolished in the *agc1*Δ*gpd1*Δ strain (Figure 4). Unlike the parental *agc1*Δ strain, *PEX34* over-expression was unable to substantially increase longevity when *GPD1* was deleted. This observation is consistent with *GPD1* participating in the *PEX34*-mediated extension of lifespan in *agc1*Δ cells.

**Figure 5:**
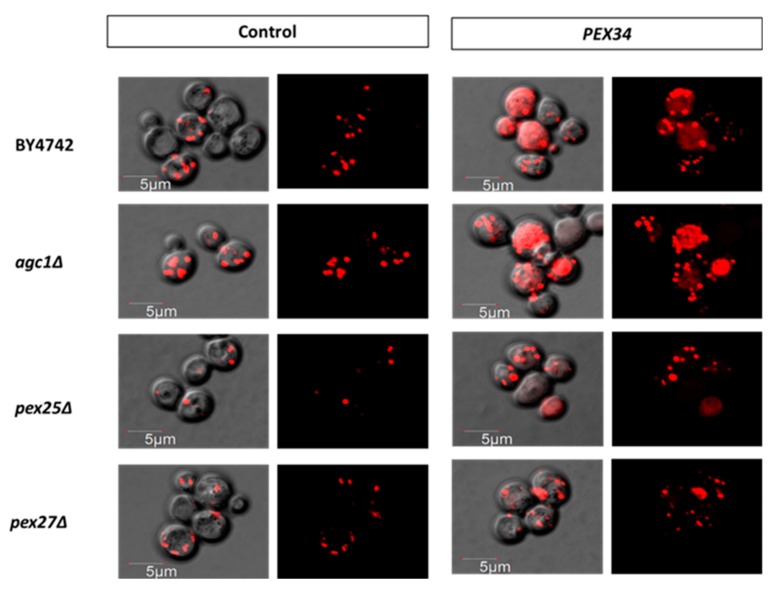
*PEX34*-induced peroxisome proliferation requires *PEX27* under a limited glucose condition. Yeasts lacking genes involved in peroxisome proliferation (*PEX25*, *PEX27*) containing peroxisome marker plasmid (RFP-PTS1) were tested for visualizing peroxisomes under *PEX34* over-expression. Yeasts were grown in SD broth supplemented with 0.1% glucose until late log phase and then used for imaging peroxisomes with confocal fluorescence microscopy. The red-dot signal represented peroxisomes. Note that *PEX34* over-expression induced the proliferation of tiny peroxisomes.

We also investigated whether the induction of peroxisome proliferation was involved in *PEX34*-mediated longevity in *agc1*Δ yeast. *PEX25* and *PEX27* encode peroxisome membrane proteins that physically interact with Pex34p and are required for *PEX34*-induced peroxisome proliferation [30]. We observed that the *PEX34*-mediated longevity of wild-type and *agc1*Δ yeasts was not reduced in the absence of either *PEX25* or *PEX27* (Figure 4). In addition, we investigated peroxisome numbers under *PEX34* over-expression in limited glucose conditions and found that *PEX34* induced peroxisome proliferation in both wild-type and *agc1*Δ yeasts. It was noticed that *PEX34* over-expression also induced the proliferation of tiny peroxisomes (Figure 5). Moreover, *PEX34* partially restored the number of peroxisomes in *pex25*Δ yeasts but failed to restore the number of peroxisomes in *pex27*Δ yeasts (Figure 5). These data confirm that the induction of peroxisome proliferation is not a primary mechanism for *PEX34*-mediated longevity.

### 3.5. Role of Malate–Oxaloacetate NADH Perosixomal shuttle in Chronological Longevity of agc1Δ Yeast

**Figure 6:**
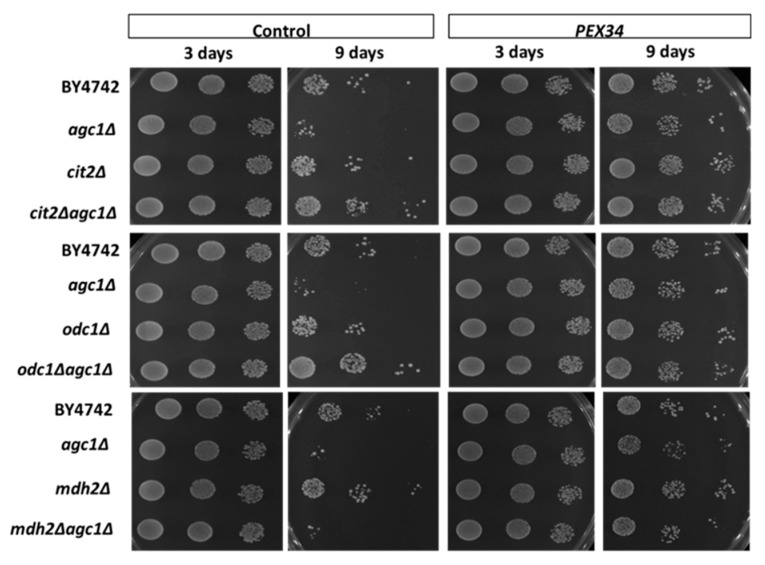
Activation of the malate–oxaloacetate NADH peroxisomal shuttle is sufficient for inducing longevity of *agc1*Δ yeasts, but the malate–oxaloacetate NADH peroxisomal shuttle is NOT essential for *PEX34*-mediated longevity in yeasts. Yeasts lacking genes disrupting the peroxisomal malate–oxaloacetate NADH shuttle (*CIT2*, *ODC1*) and genes required for this shuttle (*MDH2*) were tested for cell viability during the stationary phase under *PEX34* over-expression. The yeast array on an SD plate supplemented with 2% glucose indicated viability starting with 10^5^, 10^4^, and 10^3^ cells.

We investigated whether disruption of the malate–oxaloacetate NADH shuttle was sufficient to induce longevity of *agc1*Δ cells. *CIT2* encodes a peroxisomal citrate synthase that catalyzes the conversion of oxaloacetate and acetyl CoA to citrate and coenzyme A. *ODC1* encodes a mitochondrial oxodicarboxylate carrier, mediating exchange transport of malate and α-ketoglutarate between the cytosol and mitochondria [31,32]. It has been reported that *CIT2* and *ODC1* activity disrupt the malate–oxaloacetate NADH peroxisomal shuttle [32,33]. As seen in Figure 6, deletion of either *CIT2* or *ODC1* increased the lifespan of *agc1*Δ yeast.

**Figure 7:**
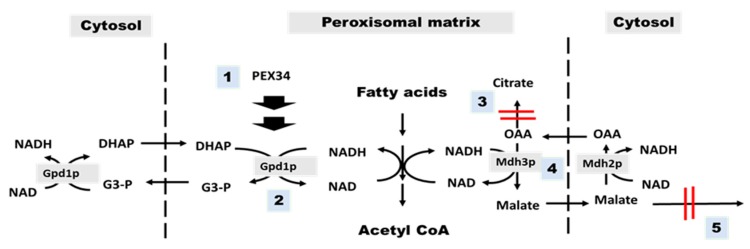
Summary of genetic mechanisms to restore the chronological longevity of *agc1*Δ yeasts. There are five genetic mechanisms for restoring the longevity of *agc1*Δ yeasts, including activation of *PEX34* over-expression (**1**), *GPD1* over-expression (**2**), deletion of *CIT2* (**3**), *MDH3* over-expression (**4**), and deletion of *ODC1* (**5**). The principle of the genetic mechanism for restoring longevity of *agc1*Δ yeasts is that induction of GPD1-mediated NAD regeneration in peroxisomes (**1**,**2**) and enhanced peroxisomal malate–oxaloacetate NADH shuttle activity (**3**–**5**). Abbreviations: Asp, aspartate; Glu, glutamate; OAA, oxaloacetate; α-KG, α-ketoglutarate, Agc1p, aspartate–glutamate carrier, Odc1p, oxodicarboxylate carrier; Mdh3p, malate dehydrogenase3; Mdh2p, malate dehydrogenase2; Cit2p, citrate synthase2; Gpd1p, glycerol 3-phosphate dehydrogenase 1; DHAP, dihydroxyacetone phosphate; G3-P, glycerol 3-phosphate.

Mdh3p and Mdh2p enzymes are components of the peroxisomal malate–oxaloacetate NADH shuttle [28]. We have examined that *PEX34*-mediated chronological longevity required *GPD1* for NAD^+^ regeneration in peroxisomes. However, deletion of *MDH3* slightly decreased the *PEX34*-mediated chronological longevity of *agc1*Δ yeasts (Figure 4). Interestingly, *PEX34* over-expression was capable of increasing longevity in the *agc1*Δ*mdh2*Δ strain (Figure 6). This indicates that *MDH2* is not required for *PEX34*-mediated longevity in *agc1*Δ cells and is consistent with increased longevity from *PEX34* over-expression from *GPD1*-mediated NAD^+^ regeneration in peroxisomes without flux through the malate–oxaloacetate NADH shuttle (Figure 7).

## 4. Discussion

The models previously used to study the pathogenesis of citrin deficiency were citrin/mitochondrial glycerol 3-phosphate dehydrogenase (mGPD) double-knockout mice and patients’ hepatocytes [1,34]. In this study, we firstly proposed *agc1*Δ yeasts to be used as a model organism to study the pathogenesis of citrin deficiency and investigated the effects of peroxisomal genes on the longevity of *agc1*Δ yeasts. The advantage of yeast in this study is its short lifespan through which we can observe chronological longevity. Moreover, yeasts contain haploid chromosomes which can easily be genetically manipulated to suit our experimental design. Although, yeasts are evolutionarily diverse from human hepatocytes and some peroxisomal genes are specific in yeast species, the basic functions in yeasts, such as fatty acid oxidation, NAD^+^ regeneration, and peroxisome proliferation, also exist in human hepatocytes. Therefore, the information from this study can provide some points to further study in citrin deficiency patients.

In our study, deletion of *AGC1*, encoding the mitochondrial aspartate–glutamate carrier, results in decreased fat utilization, decreased cytosolic NADH, and increased peroxisomal NADH signals in the stationary phase. These data suggest that loss of *AGC1* causes impaired NAD^+^ regeneration in peroxisomes resulting in decreased fatty acid oxidation with reduced production of reducing equivalents in the cytosol. These results are similar to the deletion of *GPD1*, encoding glycerol 3-phosphate dehydrogenase required for NAD^+^ regeneration in peroxisomes. Moreover, the deletion of *GPD1* also impaired fat storage at the early stationary phase. As known, Gpd1p is also required for production of glycerol, which plays role in triacylglycerol synthesis, so loss of Gpd1p results in decreased fat storage. Interestingly, deletion of *AGC1* in *gpd1*Δ cells could restore fat storage, fat utilization, and correct NADH balance in the cytosol by unknown mechanisms, which are being further investigated. Moreover, the deletion of *AGC1* results in decreased lifespan in yeasts and over-expression of *GPD1* or *MDH3*, which directly enhances NAD regeneration in peroxisomes and could restore the longevity of *agc1*Δ cells. Moreover, enhanced activity of the malate–oxaloacetate NADH peroxisomal shuttle, due to deletion of *CIT2* and *ODC1*, could restore or even enhance the longevity of *agc1*Δ cells. Collectively, it confirmed that the impaired NAD^+^ regeneration in peroxisomes is a cause of decreased chronological lifespan of *agc1*Δ cells.

*PEX34* over-expression can induce chronological longevity of *agc1*Δ as well as wild-type yeasts. *PEX34* is required for peroxisome proliferation, and over-expression of *PEX34* induces peroxisome proliferation in oleic medium, through a process that is dependent on Pex25p and Pex27p [30]. However, *PEX34*-mediated increased longevity was independent of the presence of *PEX25* and *PEX27*, but *PEX34*-mediated peroxisome proliferation required the presence of *PEX27* under a limited glucose condition. This suggests that peroxisomal proliferation is not the primary mechanism involved in promoting increased lifespan from *PEX34* over-expression. Consistent with this proposal, over-expression of *PEX11*, which can induce peroxisome proliferation in oleic medium [13], did not increase the longevity of *agc1*Δ cells. Interestingly, we demonstrated that *PEX34*-mediated longevity needs *GPD1* expression. The ability of *PEX34* to promote longevity in *agc1*Δ cells was observed in the absence of *MDH2* and *MDH3,* required for the peroxisomal malate–oxaloacetate NADH shuttle. This suggests that another mechanism is involved in *GPD1*-dependent lifespan extension from *PEX34* over-expression. Moreover, over-expression of *GPD1* itself increases the chronological lifespan of both wild-type and *agc1*Δ cells, but over-expression of *MDH3* increases the chronological lifespan in *agc1*Δ cells only, indicating that the *GPD1*-induced longevity mechanisms are not specific and may induce other mechanisms with NAD^+^ regeneration in peroxisomes. Gpd1p is required for glycerol production, which can protect cells under several stress conditions, such as osmotic stress, oxidative stress, or ER stress [35,36]. Our group has previously shown that *PEX34* over-expression results in aberrant ER morphology and induces ER stress in an acetate medium [37]. It is possible that *PEX34* over-expression also induces ER stress in the stationary phase of glucose-grown cells, resulting in the induction of *GPD1*-mediated NAD^+^ regeneration in peroxisomes. Enhanced chronological lifespan can also be mediated by the activation of mitochondrial retrograde and oxidative stress responses to overcome mitochondrial stresses [38,39]. We have previously shown that *PEX34* over-expression induced mitochondrial retrograde signaling in acetate medium [37], suggesting that *PEX34* over-expression can induce these stress responses resulting in increased longevity in yeasts.

Citrin deficiency results in intrahepatic cholestasis and fatty liver. The mechanisms of intrahepatic cholestasis and fatty liver in citrin deficiency might be involved in impaired bile acid conjugation and impaired fatty acid oxidation, which requires NAD^+^ regeneration in peroxisomes [1,7,40,41]. Administration of sodium pyruvate has the potential to increase NAD^+^ regeneration in peroxisomes, due to the presence of lactate dehydrogenases in both peroxisome and cytosolic compartments in human cells [42]. Moreover, current evidence revealed malate dehydrogenase in hepatic peroxisomes [42], indicating the presence of a peroxisomal malate–oxaloacetate NADH shuttle in humans. Based on our findings, activation the hepatic malate–oxaloacetate NADH shuttle may reduce the symptoms of citrin deficiency. Moreover, activation of peroxisome proliferator-activated receptor-alpha (PPARα) induces fatty acid oxidation in both the peroxisome and mitochondria [43]. Administration of the PPARα drug in humans might increase NAD^+^ regeneration in peroxisomes and decrease fatty liver in citrin-deficient patients, as well as restore cellular lifespan to levels seen in normal cells.

## 5. Conclusions

Peroxisomal dysfunctions observed using the yeast model for citrin deficiency appear to result in decreased cellular longevity. Processes that enhance peroxisomal NAD^+^ regeneration, either directly or indirectly, can increase chronological lifespan in *agc1*Δ yeast (Figure 7). Enhancing peroxisome function or inducing NAD^+^ regeneration in peroxisomes are suggested for further study in patients’ hepatocytes.

## Supplementary Materials

The following are available online at https://www.mdpi.com/2079-9721/8/1/2/s1, Table S1: Primer names and their sequences used in this study, Table S2: Yeast genotypes used in this study.
