# Supplementary material for: Genetic Analysis of Peroxisomal Genes Required for Longevity in a Yeast Model of Citrin Deficiency
Source: Diseases. 2020 Jan 9;8(1):2. doi: 10.3390/diseases8010002 (PMC7151034; doi:10.3390/diseases8010002)
Supplement: Supplementary file 1 [file diseases-08-00002-s001.pdf]

Table S1. Primer names and their sequences used in this study

AGC1_BamH1_US5'	GATGACAGCAATGGTGGATCCATTCTAGGCAATACTG
AGC1_Sal1_US3'	GAAGGGATCAGGCTACGTCGACAATTATGGCAAACGCG
AGC1_NotI_DS5	CAATTTGGGTTCACTGCGGCCGCCTATGAATTATTCAAGGGC
AGC1_BamHI_DS3	CTGTATGTAGGCTCGATTGAGTGGGCGGC
PEX34_BamHI-5	GTAAAGGAAGAAGAAAAGGATCCGGAATGGTTTCGAAG
PEX34_XbaI-3	CTTACAGAACTAAATCGTTCTAGAATAAAATTATAAATTATTC
PEX5_BamH1-10_5	ATATACATCAATAAAGGATCCATCATAACACATGGACGTAGG
PEX5_SalI_1843_3	GAACATAAAATTGCGGTCGACCATATCAAAACGAAAATTCTCC
PEX11_BamHI-5	CTTCATCAAGTAATAGTGGATCCAATATGGTCTGTG
PEX11_SpeI-3	CCAGATAGCAACAAGAGATGACTAGTAAGCTATGTAGCTTTCCAC
MDH3_BamHI-6_5	AATAAAAAGAGACAAAGGATCCTAAACATGGTCAAAGTCGC
MDH3_HindIII_1042_3	GAGTTAAGAAAAATATAAAGCTTGAAGTAGCTCATAGCTTGG
GPD1_BamHI-7_5	CACAAACACAAATATGGATCCTATAAAGATGTCTGCTGCTGC
GPD1_HindIII_1189_3	GAAAGTATGATATGTAAGCTTTCTCCAATAAATCTAATCTTCATG TAG
NDE2-3_SalI_1684	GAATTGGAACAATGAGTCGACAAAACAAGGACTACACGGAG
NDE2-5_BamHI_-7	CAACCACCGTACTTGGGATCCGTATAGATGCTGCCCAGAC

Table S2. Yeast genotypes used in this study

Yeast strain	Genotype
BY4742	*MATα*, *his3Δ1*, *leu2Δ0*, *lys2Δ0*, *ura3Δ0*
pex25Δ	*MATα, his3Δ1, leu2Δ0, lys2Δ0, ura3Δ0, pex25::KanMX4*
pex27Δ	*MATα, his3Δ1, leu2Δ0, lys2Δ0, ura3Δ0, pex27::KanMX4*
cit2Δ	*MATα, his3Δ1, leu2Δ0, lys2Δ0, ura3Δ0, cit2::KanMX4*
odc1Δ	*MATα, his3Δ1, leu2Δ0, lys2Δ0, ura3Δ0, odc1::KanMX4*
mdh3Δ	*MATα, his3Δ1, leu2Δ0, lys2Δ0, ura3Δ0, mdh3::KanMX4*
gpd1Δ	*MATα, his3Δ1, leu2Δ0, lys2Δ0, ura3Δ0, gpd1::KanMX4*
agc1Δ	*MATα, his3Δ1, leu2Δ0, lys2Δ0, ura3Δ0, agc1::HIS3*
pex25Δ agc1Δ	*MATα, his3Δ1, leu2Δ0, lys2Δ0, ura3Δ0, pex25::KanMX4, agc1::HIS3*
pex27Δ agc1Δ	*MATα, his3Δ1, leu2Δ0, lys2Δ0, ura3Δ0, pex27::KanMX4, agc1::HIS3*
cit2Δ agc1Δ	*MATα, his3Δ1, leu2Δ0, lys2Δ0, ura3Δ0, cit2::KanMX4, agc1::HIS3*
odc1Δ agc1Δ	*MATα, his3Δ1, leu2Δ0, lys2Δ0, ura3Δ0, odc1::KanMX4, agc1::HIS3*
mdh2Δ agc1Δ	*MATα, his3Δ1, leu2Δ0, lys2Δ0, ura3Δ0, mdh2::KanMX4, agc1::HIS3*
mdh3Δ agc1Δ	*MATα, his3Δ1, leu2Δ0, lys2Δ0, ura3Δ0, mdh3::KanMX4, agc1::HIS3*
gpd1Δ agc1Δ	*MATα, his3Δ1, leu2Δ0, lys2Δ0, ura3Δ0, gpd1::KanMX4, agc1::HIS3*
